# The Neglect of Recent Research on the Influence of the Cerebellum on Auditory Processing

**DOI:** 10.1007/s12311-026-02004-3

**Published:** 2026-05-02

**Authors:** José Mário Prati

**Affiliations:** Poços de Caldas, Minas Gerais Brazil

**Keywords:** Cerebellum, Auditory Processing, Cerebellar Cortex, Auditory Pathways

## Abstract

Classic studies beginning in the 1940s started to demonstrate, in animals, activation of different cerebellar regions following the delivery of auditory stimuli. Stimulation of these cerebellar regions elicits responses in other brain regions known to be involved in auditory processing. Lesions produced in specific cerebellar regions and the use of molecular markers suggest bidirectional connectivity between the cerebellum and the cochlear nucleus. Although such evidence exists, the findings are highly heterogeneous with respect to the vermal and cerebellar hemispheric regions potentially involved in auditory processing. In addition, the evidence is supported predominantly by animal studies, whereas studies in humans are highly scarce and inconclusive. As a result, numerous gaps remain regarding the involvement of the cerebellum in auditory processing, as well as its connectivity and the effects of these connections on other encephalic structures that also participate in this processing. Given the lack of recent studies on the influence of the cerebellum on auditory processing, there is a neglect in the study of this function, which has potential as a field of research.

## Introduction

The discoveries and understanding of the participation and influence of the cerebellum in the processing of multiple known functions occur especially through the identification of anatomical and metabolic substrates demonstrating structural and functional connectivity of the cerebellum with several areas of the nervous system. Previous studies in humans and animals demonstrate structural and functional connectivity of the cerebellum with the motor cortex [1], parietal cortex [2, 3], temporal cortex, and prefrontal cortex through the cerebello–thalamo–cortical and cortico–ponto–cerebellar pathways [4].

The participation and influence of the cerebellum in the processing of certain functions have been extensively studied, such as emotion [5–7], cognition [8, 9] and autonomic functions [10, 11], however other functions such as hearing have not received similar attention. Studies dating back to the 1940s using cats and rodents had already observed cerebellar activation in response to auditory stimulation. In humans, neuroimaging studies have demonstrated cerebellar activation during the performance of verbal memory tasks [12] and semantic processing of words [13].

Therefore, initial evidence provided clues about a possible involvement of the cerebellum in auditory processing. Over the years, new studies have emerged and strengthened this view. However, the real role of the cerebellum in auditory processing remains obscure. Technological and research advances have allowed studies to evolve, especially clinical studies. This has made it possible to identify clinical manifestations related to sensory and spatial contexts of auditory stimuli in individuals with cerebellar lesions. Therefore, the aim of this review is to identify the cerebellar regions involved, the cerebellar connections with brain structures, and possible roles of the cerebellum in auditory processing.

## Methods

Searches were conducted in PubMed and Google Scholar using search terms such as “cerebellum,” “auditory function,” “auditory processing,” and “cerebellar auditory processing,” employing Boolean operators AND and OR. Preclinical and clinical studies addressing the topic of this review were analyzed, and relevant information was collected on the cerebellar regions activated in response to auditory stimuli and potential pathways of cerebellar connectivity with other brain structures known to be involved in auditory processing.

## Results

### Evidence from Preclinical Studies of Cerebellar Involvement in Auditory Processing

Snider and Stowell (1944) [14] were among the first to demonstrate that auditory stimulation with a loudspeaker click elicited electrical potentials with more robust responses in the lobulus simplex and in the anterior portion of the tuber vermis, and less robust responses in the caudal part of the tuber vermis of the cerebellum in anesthetized cats. In rodents, electrical potentials were identified in the lateral half to one third of Crus IIB [15]. Subsequently, Gacek (1973) [16] demonstrated that lesions made in lobules VIII and IX of the cerebellar vermis and in lobule X produced patterns of degeneration that extended to the posteroventral cochlear nucleus, suggesting descending projections from the cerebellum to the cochlear nucleus. Additionally, another study demonstrated labeled cells in pontine nuclei after injection of anterograde tracer (PHA-L) into anteroventral and posteroventral cochlear nuclei, suggesting a potential pathway connecting the cochlear nuclei and cerebellum [17]. Finally, Huang and colleagues (1982) [18] demonstrated that injections of the enzyme horseradish peroxidase (HRP) into lobules VI, VII, and VIIIA of the cerebellar vermis in cats elicited significant cell labeling in the posteroventral cochlear nucleus and diffuse labeling in the dorsal and anteroventral cochlear nuclei, bilaterally. Conversely, HRP injections into the paravermal region and the cerebellar hemisphere resulted in reduced cell labeling in the cochlear nucleus, indicating that the auditory region of the cerebellum corresponds to the vermal lobules of the posterior lobe of the cerebellum. These findings suggested that the cerebellum may have anatomical connectivity with the cochlear nucleus, the main target of the vestibulocochlear nerve, which carries electrical potentials originating from the cochlea.

Teramoto and Snider (1966) [19] demonstrated that electrical stimulation of the tuber vermis in cats elicited responses in the inferior colliculus, medial geniculate nucleus, and primary and secondary auditory cortices ipsilateral to the cerebellar stimulation, suggesting that the cerebellum may send projections to primary auditory processing nuclei as well as to higher centers of secondary processing. Stimulation of the anterior vermal region or the lobulus simplex was shown to elicit an inhibitory effect on cochlear microphonics and on the action potential of the auditory nerve in guinea pigs [20], providing an initial indication of one of the cerebellum’s functions in auditory processing.

Although sensitivity to 1 kHz sound frequencies has been demonstrated in neurons of vermal lobules VIIA and VIIB [21], other studies have shown that sound frequency sensitivity does not vary according to location within lobules VI and VII [22], and that some neurons in vermal lobules VI and VII exhibit selective sensitivity to interaural differences in intensity and time and to the movement of the sound source [23]. Furthermore, cerebellar neurons show asymmetrical response activity, indicating a preference for the movement of the sound source, while other neurons show symmetrical response activity, indicating indifference to the direction of movement of the sound source [24]. This suggests that the cerebellum may have an important role in identifying the location, movement, and direction of sound.

**Fig. 1:**
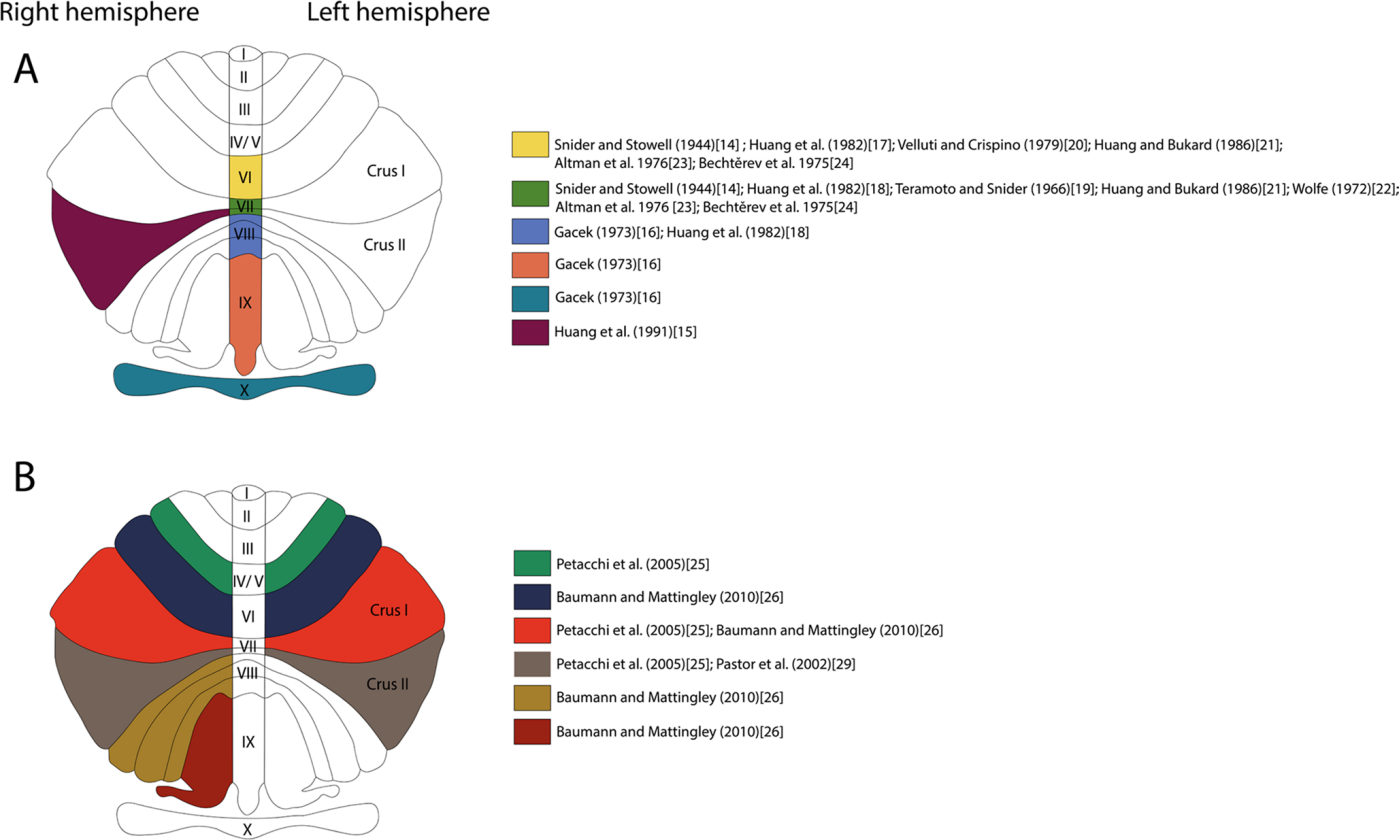
Cerebellar regions of the vermis, hemispheres, and flocculonodular lobe, which have been observed to have some involvement in auditory processing in previous studies. **A** Findings from preclinical studies on the potential involvement of the vermal lobules, right Crus II, and flocculonodular lobe in auditory processing. **B** Findings from clinical studies of cerebellar regions, especially cerebellar hemispheres, that demonstrated activity in the processing of auditory stimuli. Created with Adobe Illustrator

Observing the findings of preclinical studies, it is possible to identify a consensus that regions of the posterior lobe of the cerebellum, such as vermal lobules VI, VII, and VIII, appear to be more involved in auditory processing (Fig. 1). Furthermore, experiments show that these regions may exhibit structural and functional connectivity with the cochlear nucleus, inferior colliculus, thalamus, and auditory cortex; however, the functional influence of the cerebellum on these structures is still unknown. Finally, studies demonstrating responses of cerebellar neurons to the movement of a sound source have provided a valuable finding that can be investigated to establish a correlation between the potential role of the cerebellum in identifying the location and direction of sound and its influence on other brain structures involved in auditory processing.

### Evidence from Clinical Studies of Cerebellar Involvement in Auditory Processing

As in classic preclinical studies, clinical studies focused on identifying cerebellar regions that showed some activity in response to auditory stimuli. Studies that delivered different types of auditory stimuli during passive listening and active listening demonstrated cerebellar activation in left hemispheric Crus I, bilateral hemispheric lobule V, and posterior regions of right hemispheric Crus I and Crus II, with the highest activation peak in right hemispheric Crus I [25]. In another study, it was demonstrated that processing auditory stimuli with a moving sound source elicited increased BOLD activity in bilateral hemispheric lobules VI and in right hemispheric lobules VIIIA and VIIIB. Additionally, increased neural activity was observed in left Crus I associated with a reduction in the strength of auditory stimulus movement, and increased neural activity in right lobule IX and right Crus I associated with an increase in the strength of auditory stimulus movement [26]. Therefore, while preclinical studies have demonstrated that vermal lobules showed the most prominent signs of involvement in auditory processing, clinical studies have demonstrated that hemispheric regions of the cerebellum may also be involved in this processing (Fig. 1). In addition, activity responding to movement of the sound stimulus source was also observed, further reinforcing the potential role of the cerebellum in identifying the location and direction of sound.

**Fig. 2:**
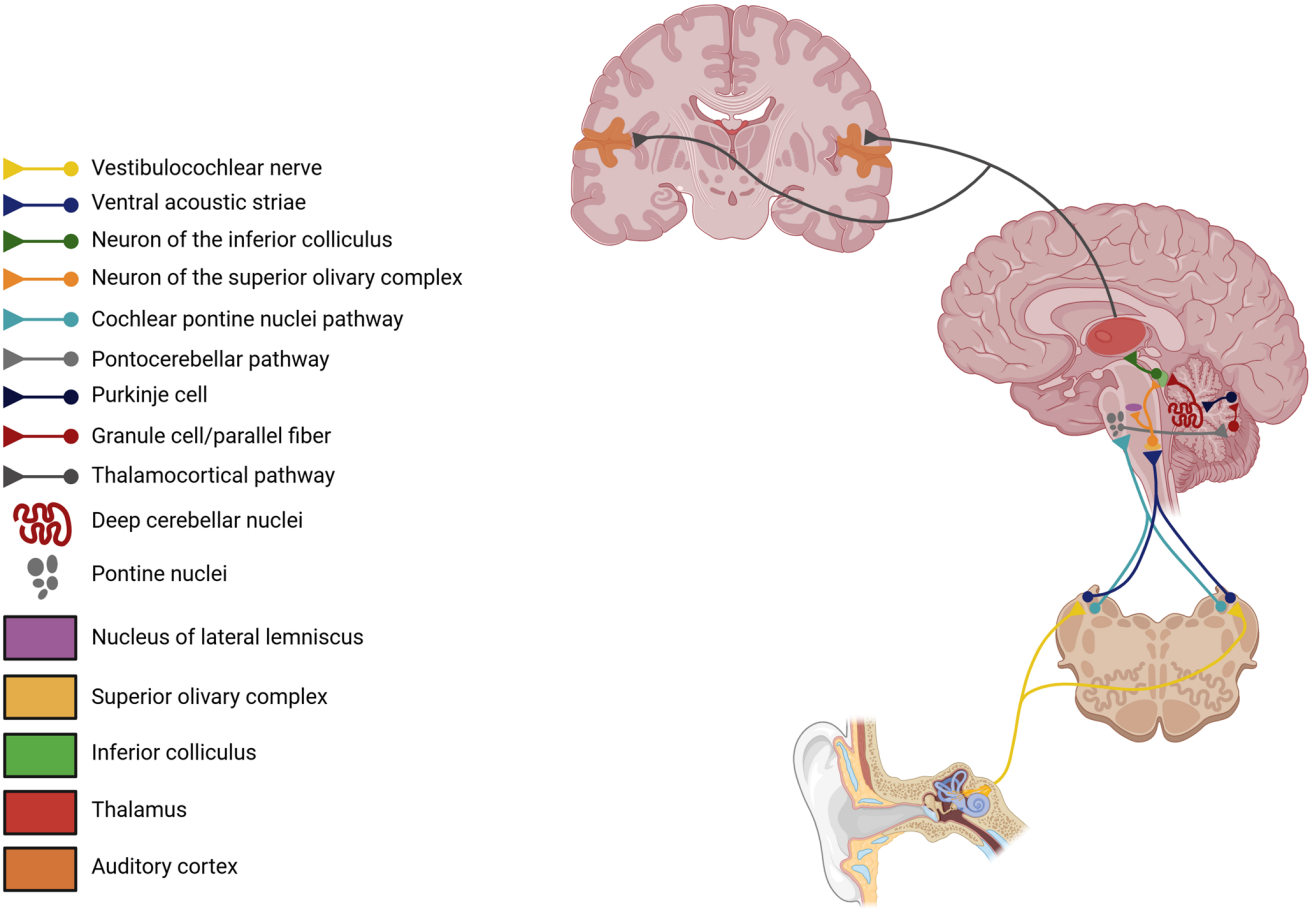
Potential afferent pathways of auditory processing, including afferent projections to the cerebellum originating from the cochlear nuclei, reaching the cerebellum through the pontine nuclei; and efferent projections from the cerebellum to the inferior colliculi. The cerebellum can also send efferent projections to the cochlear nuclei, constituting a bidirectional connecting pathway (not shown). The intermediate acoustic stria and dorsal acoustic striae have been omitted from this figure. Created with BioRender

Regarding connectivity, strong functional connectivity has been demonstrated between the hemispheric lobule VI and the superior temporal cortex at rest [27]. Different regions of the superior temporal gyrus (STG), including Heschl’s gyrus, planum temporale, and lateral STG, have significant functional coupling with different cerebellar regions considering the interindividual variability of functional connectivity (FC) of the auditory cortex. The lateral STG has been shown to have strong FC with the contralateral cerebellar hemisphere and Heschl’s gyrus, and the planum temporale has strong FC with both cerebellar hemispheres. Greater functional coupling of lobules VIIIA, VIIIB, and lobules I-IV/V of both cerebellar hemispheres with planum temporale than with the lateral STG has also been demonstrated, as well as greater functional coupling of Crus I, Crus II, and the left VIIIA hemispheric lobule with the lateral STG than with the planum temporale. These findings indicate that connectivity between the auditory cortex and the cerebellum occurs through distinct cortico-cerebellar pathways due to the level of variability in the auditory cortex [28]. Figure 2 illustrates the potential communication pathways between the cerebellum and cortical and subcortical structures involved in auditory processing.

Offering clicks at 40 Hz triggers increased cerebellar metabolism in the posterior cortical portion of both cerebellar hemispheres, especially in the contralateral hemisphere in relation to auditory stimulation [29] and activation of the coupling between the STG/superior temporal sulcus and Crus II compared to stimulation at 12 and 26 Hz, evidencing a selective modulation of this connectivity dependent on the stimulus frequency [30]. The findings of preclinical and clinical studies diverge with respect to frequency sensitivity in the activation and connectivity of the cerebellum with the auditory cortex. Different cerebellar regions may exhibit greater sensitivity to different frequencies, and this may impact the influence that the cerebellum can exert on subcortical structures involved in auditory processing, such as the cochlear nuclei and inferior colliculi.

### Auditory Processing Impairments Caused by Cerebellar Lesions

Cerebellar lesions can cause auditory disorders including hearing loss and tinnitus. Individuals with chronic cerebellar lesions exhibit impairments in the Synthetic Sentence Identification test (SSI), suggesting an inability to select auditory information [31]. A case report described a patient with cerebellar gangliocytoma who presented with fluctuating hearing loss and tinnitus [32]. A high incidence of individuals diagnosed with stroke in the anterior inferior cerebellar artery presents with hearing loss [33]. Under pathological conditions, alterations occur in behavioral tests and cerebellar FC. In a study involving children with different types of cerebellar tumors located in vermal as well as right or left hemispheric regions, reduced auditory memory performance was observed in children with cerebellar tumors compared with children without the oncological condition [34].

A more recent study revealed important implications of auditory sensory deprivation on intra-cerebellar FC, including reduced FC between the right hemispheric lobule VIIB and right Crus I, and between the cerebellum and other brain regions, including reduced FC between the left hemispheric lobule VI and the left temporal pole, supramarginal gyrus, and insula; reduced FC between the right hemispheric lobule VI and the left inferior frontal gyrus; and reduced FC between the left hemispheric lobule VIIB and the left middle frontal gyrus and bilateral thalamus in individuals with moderate to severe bilateral hearing loss. Furthermore, a negative correlation was demonstrated between FC of the left hemispheric lobule VI and the left insula and scores on the Self-Rating Anxiety Scale (SAS); a negative correlation between FC of the left hemispheric lobule VI and the left temporal pole with hearing loss scores and SAS scores; and a negative correlation between FC of the left hemispheric lobule VI and the left inferior frontal gyrus with hearing loss severity and SAS scores [35].

Individuals with sudden sensorineural hearing loss (SSNHL) on the right showed impaired FC in the right lobule V with mean time series of the cerebellar network, also demonstrating a negative correlation with the SAS score. A reduction in outflow from the right lobule V to the STG and to frontal lobe regions was also observed compared to control individuals, and an increase in FC with the right middle frontal gyrus, the latter possibly being a predictive factor for anxiety in individuals with SSNHL. In individuals with long-term sudden sensorineural hearing loss (RLSNHL), there was an increase in FC in the right lobule V with mean time series of the cerebellar network, demonstrating a negative correlation with performance on the symbol digit modalities test (SDMT), and greater effectiveness of FC in the right insula and temporal pole with the right lobule V [36]. These findings indicate that the cerebellum may be importantly involved not only in auditory sensory processing, but also in emotional and cognitive implications potentially related to auditory sensory deprivation.

### Clinical Implications of Cerebellum’s Involvement in Auditory Processing

Considering the findings presented, it becomes possible to raise some hypotheses about the clinical implications of the cerebellum as a component of auditory processing. The cerebellum has been shown to act in the identification of movement and direction of auditory stimuli, as demonstrated by preclinical studies [23, 24] and clinical study [26]. Studies involving individuals with cerebellar lesions have demonstrated hearing impairments and the presence of tinnitus [32, 33], as well as cognitive and affective repercussions [34–36], but have not evaluated the outcome involving binaural hearing. Therefore, future studies could investigate whether individuals with cerebellar lesions present impairments in the identification of movement and direction of auditory stimuli.

The cerebellum may play a role in cognitive abilities involving hearing and in the emotional repercussions of auditory sensory deprivation. Studies show that auditory sensory deprivation impairs information processing speed and neuropsychological state, potentially inducing anxiety disorders. Using the cerebellum as a therapeutic target could contribute to improving these outcomes. The use of non-invasive cerebellar neuromodulation techniques could be a viable approach, considering that studies investigating the effectiveness of these interventions in individuals with sensory deprivation are lacking.

## Conclusion

Unfortunately, most studies investigating the participation or influence of the cerebellum in auditory processing are scarce, dated and present heterogeneous results, demonstrating that this line of research has not received significant attention and has therefore been neglected in recent years. The delivery of auditory stimuli elicits cerebellar activation, but the cerebellar regions possibly involved in auditory processing are still not fully known, especially considering the divergence between preclinical and clinical studies on the cerebellar regions involved in auditory processing and the lack of knowledge about the involvement of the deep cerebellar nuclei. Additionally, it is not completely known either the cerebellar connectivity with other brain structures that process auditory stimuli; the effect of cerebellar connectivity on these structures; the role of the cerebellum in cognitive processing related to hearing; and possible structural and functional characteristics of the cerebellum in individuals of different ages and pathological conditions with respect to auditory processing. Future studies may address these questions and effectively advance the understanding of the cerebellum’s involvement in auditory processing.

## Data Availability

All data described are derived from the studies cited.
